# Effect of NT-proBNP on Serum Calcium: A Longitudinal Analysis

**DOI:** 10.3390/medicina61040755

**Published:** 2025-04-19

**Authors:** Maria Rita Stancanelli, Giuseppe Restivo, Thea Corriere, Carmela Cannarozzo, Maria Gabriella Ferrara, Rosario Salemi, Maria Eva Sberna, Angelo Iraci, Ada Restivo, Valeria Furia, Elisa Longhitano, Domenico Santoro, Vincenzo Calabrese

**Affiliations:** 1 Unit of Nephrology and Dialysis, Department of Medicine and Surgery, Hospital of Enna “Umberto I”, 94100 Enna, Italy; mariarita.stancanelli@gmail.com (M.R.S.); gi.res@tiscalinet.it (G.R.); theacorriere@gmail.com (T.C.); carmen.cannarozzo@virgilio.it (C.C.); mgaferrara@gmail.com (M.G.F.); rosario.salemi@hotmail.com (R.S.); mariaevasberna@gmail.com (M.E.S.); 2 Faculty of Medicine and Surgery, University Dunarea de Jos, 94100 Enna, Italy; angelo.iraci2001@gmail.com; 3 Department of Service, Hospital of Enna “Umberto I”, 94100 Enna, Italy; ada.restivo@asp.enna.it (A.R.); furiavaleria@yahoo.it (V.F.); 4 Unit of Nephrology and Dialysis, Department of Clinical and Experimental Medicine, University Hospital “G. Martino”, 98100 Messina, Italy; elisa.longhitano@libero.it (E.L.); dsantoro@unime.it (D.S.); 5 Unit of Nephrology and Dialysis, Department of Medicine and Surgery, University of Enna “Kore”, 94100 Enna, Italy

**Keywords:** BNP, calcium, eGFR, electrolytes, NCX

## Abstract

*Background and Objectives*: Brain natriuretic peptide (NT-proBNP) is a biomarker widely used in diagnosing and monitoring heart failure. Its impact on electrolyte homeostasis is known, particularly for sodium. However, its relationship with serum calcium remains unclear. This retrospective observational study aimed to investigate the longitudinal association between NT-proBNP and serum calcium levels in a cohort of hospitalized patients with the goal of determining whether NT-proBNP could have a direct or indirect impact on calcium metabolism. *Materials and Methods*: We included 688 patients with 1022 repeated measurements of NT-proBNP and serum calcium collected during hospitalization from March 2022 to February 2025. Linear mixed models (LMMs) were employed to analyze longitudinal associations, adjusting for age, eGFR, estimated plasma volume status (ePVs), CRP, potassium, and albumin. *Results*: Baseline analysis revealed a negative correlation between NT-proBNP and serum calcium (r = −0.23, *p* < 0.001). Univariate LMM demonstrated a significant negative association (β = −1.3 × 10^−5^, *p* < 0.001), which remained significant in multivariate analysis (β = −6.9 × 10^−6^, *p* = 0.01), accounting for intrasubject variability. This suggests that as NT-proBNP increases, serum calcium levels decrease within individual patients, independent of confounders. This study’s findings indicate that NT-proBNP may influence calcium excretion, possibly through mechanisms involving the sodium–calcium exchanger (NCX) in renal tubules, similar to its effects on sodium homeostasis. *Conclusions*: This is the first study to evaluate the longitudinal impact of NT-proBNP on serum calcium, highlighting a potential clinical relevance in patients with cardiac dysfunction. Limitations include a retrospective design and a lack of urine calcium data. Further research is warranted to validate these findings and elucidate the underlying mechanisms.

## 1. Introduction

Brain natriuretic peptide (BNP), primarily secreted by cardiomyocytes after myocardial stretch, was initially identified in porcine brain cells. In humans, this peptide is composed of 32 amino acids and represents the biologically active form of NT-proBNP.

BNP plays a crucial role in regulating blood pressure and maintaining hydroelectrolytic balance, exerting both cardioprotective and nephroprotective effects. Clinically, BNP is a valuable biomarker for diagnosing and monitoring heart failure, and its circulating levels correlate directly with the severity of the condition. Myocardial stretch, elevated angiotensin II levels, and sympathetic nervous system-mediated vasoconstriction stimulate its secretion. Physiologically, BNP needs to bind to natriuretic peptide receptor A (NPR-A) to activate the cyclic guanosine monophosphate (cGMP) signaling pathways. BNP’s cardiovascular effects include coronary vasodilation, which enhances myocardial perfusion, as well as anti-hypertrophic and anti-fibrotic properties. Additionally, BNP inhibits sympathetic nervous system activity, reducing catecholamine release, tachycardia, and vasoconstriction. Consequently, it decreases both preload and afterload, exerting beneficial effects on heart failure pathophysiology [1]. Furthermore, BNP acts as an antagonist of the RAAS and SNS systems and is also involved in electrolyte homeostasis [2].

The correlation between BNP and sodium is well known due to its natriuretic action. Under physiological conditions, BNP stimulates diuresis and sodium excretion to reduce blood volume, leading to hyponatremia. This is associated with hypervolemic hyponatremia in severe heart failure, contrasting with the excessive fluid retention in heart failure [3].

BNP also stimulates renal chloride excretion related to sodium excretion through tubuloglomerular feedback. Low blood chloride levels detected by the macula densa trigger the production of renin, angiotensin, and aldosterone. The RAAS stimulates the SNS-mediated afferent arteriole constriction, which leads to reduced renal perfusion, natriuresis, and diuresis. Furthermore, an increased tubular reabsorption of chloride occurs via co-transport with sodium, resulting in fluid retention [4].

A direct correlation between BNP and potassium has not been described in the literature, but an indirect link emerges in heart failure. A study reported low serum potassium levels in acute heart failure patients treated with exogenous ANP (known as Carperitide) within the first 48 h, either as monotherapy or in combination with furosemide, while sodium levels were unchanged. Considering that ANP and BNP act through the same NPR-A—cGMP pathway, a similar effect could be hypothesized for BNP [5].

An observational study showed a direct association between ANP and urinary calcium excretion. However, to our knowledge, no study has evaluated its impact on serum calcium. Our analysis aims to analyze the impact of NT-proBNP on serum calcium longitudinally, considering intrasubject variability.

## 2. Materials and Methods

The study is in conformity with the guidelines of the Italian Data Protection Authority and in agreement with the Declaration of Helsinki. Data were anonymously and retrospectively extracted, and no relationship between patients and laboratory values was retrieved. All admitted patients signed written consent at the time of admission.

### 2.1. Study Population and Laboratory Data

In this cohort study, we included patients admitted to our hospital, in which NT-proBNP is evaluated as normal clinical practice, from 1 March 2022 to 28 February 2025. NT-proBNP and serum calcium measurements were collected during the hospitalization. 

Patients had a median number of 2 measurements during this time, ranging from 1 to 16. Of the 1253 patients who comprised the original cohort, only for 688 patients, for a total of 1022 repeated measurements, did the data include both serum calcium and NT-proBNP at the same time; these patients were included in the analysis. The age, sex, and clinical and biochemical characteristics of the study population are detailed in Table 1.

### 2.2. Data Collection

Laboratory and clinical data were collected locally from the central lab of our hospital. Laboratory data included serum calcium, hemoglobin (Hb), hematocrit (Hct), white blood cells (WBCs), platelets (PLTs), C-reactive protein (CRP), sodium, potassium, albumin, serum urea, serum creatinine, and estimated glomerular filtration rate (eGFR, computed according to the CKD-EPI formula). Estimated plasma volume status (ePVs) was computed according to the formula 100 × (1 − Hct)/(Hb).

### 2.3. Statistical Analysis

Data were described as mean ± standard deviation, median and interquartile range, or proportion, as appropriate. Calcium and NT-proBNP, as well as quantitative confounders, were included in the analysis as continuous variables. The distribution of the variables was investigated by the Kolmogorov–Smirnov test followed by graphic evaluation. The number of missing data varied across variables. In detail, albumin was missing in about 55% of measures, WBC in 3.1%, potassium in 3.7%, sodium in 0.6%, Hct in 3.1%, creatinine in 1.1%, eGFR in 1.1%, and serum urea in 5.3%. Other variables included in the multivariate models had less than 0.01% of missing data. Missing values were neither related to the center that provided the data nor to specific characteristics of the patients and so were considered at random. As mixed-effects models are well equipped to handle missing (at random) response data if estimated using likelihood methods, we did not impute or recover them.

The baseline correlation between serum calcium and NT-proBNP was tested through Pearson’s correlation coefficient. The longitudinal association between NT-proBNP and serum calcium was analyzed by univariate and multivariate linear mixed models (LMMs) for repeated measures. In adjusted analyses, we included all variables related to serum calcium and NT-proBNP with a *p*-value < 0.1 as potential confounders. Multivariate models adjusted for age, eGFR, ePVs, CRP, potassium, and albumin were evaluated through the analysis of residuals. Relationships with a *p*-value < 0.05 were considered significant.

## 3. Results

The clinical, demographic, and somatometric characteristics of the entire population are summed up in Table 1. The mean age was 79 ± 12 years. Hemoglobin had a normal distribution with a mean value of 11.3 ± 2.3 g/dL. Serum calcium was 8.5 ± 0.9, ranging from 4.8 to 12.3 mg/dL, and NT-proBNP distribution was not Gaussian, with a median of 3820 (1013–12,258) (Table 1).

**Figure 1:**
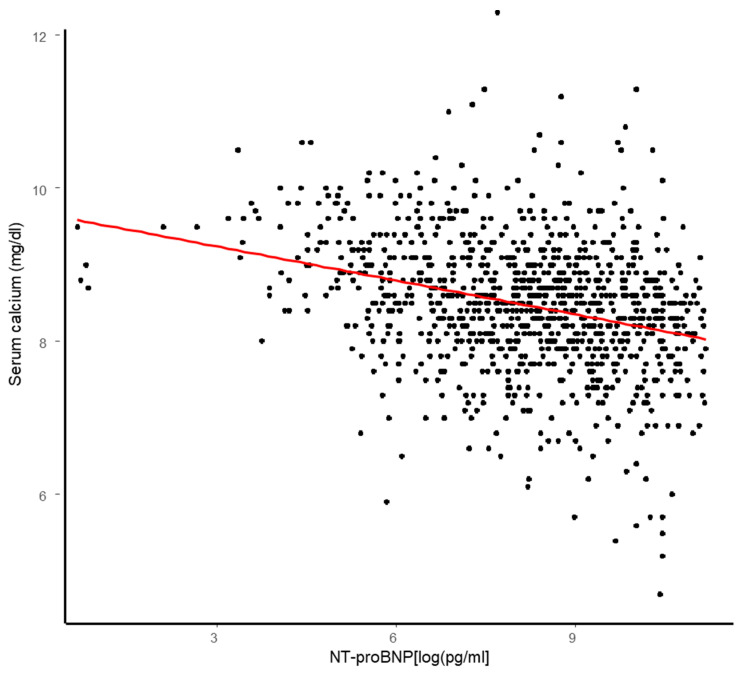
Association between NT-proBNP (log-transformed) and serum calcium. Dots represent each value, whereas red line represents the association trend.

Serum calcium and NT-proBNP levels were negatively related (r = −0.23, *p* < 0.001) (Figure 1), and both variables were related clinically or statistically to age, albumin, CRP, eGFR, ePVs, or potassium (all *p* < 0.1).

Univariate linear regression models showed a negative association between calcium and NT-proBNP (β = −1.72 × 10^−6^, *p* < 0.001), and the multivariate analysis showed a trend of significance, and the direction of the relationship was the same (β = −6.1 × 10^−7^, *p* = 0.10). 

Conversely, adjusting for intrasubject variability with a model for repeated measures, the negative association between serum calcium and NT-proBNP was confirmed both in the univariate and multivariate linear mixed model (β = −1.3 × 10^−5^, *p* < 0.001; Adjβ = −6.9 × 10^−6^, *p* = 0.01) (Table 2) (Figure 1). Albumin, ePVS, and serum potassium were the other variables related to serum calcium, according to our multivariate linear mixed model.

## 4. Discussion

Our analysis found a negative association between NT-proBNP and serum calcium. In detail, we found a trend of significance in the relationship between serum calcium and NT-proBNP when intrasubject variability was not tested. A significant association was found when intrasubject variability was included in the model. This confirms that NT-proBNP could impact the serum calcium level, indicating that the calcium level changes in association with NT-proBNP changes in individual patients. The difference between the linear mixed model and linear regression can be delegated to different baseline NT-proBNP values in the subjects due to the high variability of our sample. Indeed, our analysis showed that NT-proBNP changes are related to calcium variation when the model was adjusted for individual patients, independently of hemoconcentration, albumin, age, and renal function. To our knowledge, this is the first study that evaluates the effect of NT-ptoBNP changes on serum calcium, evaluating their slope modification in time.

In detail, for each unitary increase in NT-proBNP, calcium decreased by 7 × 10^−7^ mg/dL. Although this value seemed to have no clinical impact, the large variation shown in NT-proBNP usually varies from a few dozen to tens of thousands. Thus, a unitary change on a large scale reflects important changes in calcium concentration.

Even though the pathogenetic process is unclear, NT-proBNP seems to increase calcium excretion. Indeed, plasma ANP is associated with the escape phenomenon in patients with reduced sodium excretion, with an increase in urinary calcium [6]. In detail, Rossi et al. showed a reduction in ionized calcium in patients with aldosteronism, relating it to expansion volume and ANP production, more than to direct channel activity. The increased urinary calcium seems to be due to a reduced reabsorption of calcium caused by reduced sodium reabsorption at proximal tubular sites, as well as due to mechanisms of increased calcium excretion via reduced calcium reabsorption in the distal tubule [7].

BNP also plays a pivotal role in the heart–kidney axis. By activating NPR-A and increasing cGMP levels, BNP inhibits sodium-dependent channels in the collecting duct, thereby promoting natriuresis and diuresis. This natriuretic and diuretic effect is further potentiated by the inhibition of renin and aldosterone secretion, leading to reduced sodium and water reabsorption in the distal tubule and collecting duct. These mechanisms contribute to the regulation of blood volume and blood pressure. Furthermore, BNP induces afferent arteriole vasodilatation and efferent arteriole constriction, resulting in enhanced glomerular filtration and renal perfusion [8].

BNP influences the homeostasis of calcium-regulating proteins. Notably, BNP stimulates the sodium–calcium exchanger (NCX) through increased cGMP levels. NCX channel allows three sodium ions to inflow in exchange for one calcium ion. A key NCX characteristic is its bidirectional ion transport: calcium is usually expelled, but during depolarization, the transporter reverses its activity, drawing calcium into the cytoplasm while extruding sodium. BNP may alter NCX activity and reverse sodium–calcium exchange in the myocardium, modifying its intracellular and extracellular concentrations. NCX is also located in the renal tubule. Thus, it is likely that a similar process happens in the renal distal tubule. It would be interesting to determine whether NCX behaves similarly in the kidney and whether it increases calciuria [9].

Although the active form of calcium is ionized calcium, our study aimed to analyze whether NT-proBNP could have an impact on total calcemia as an indirect consequence of calciuria [10].

However, as principal features clinically related to serum calcium concentration, we included albumin, renal function decline, and ePVs in our models. Indeed, albumin is well known for linking about 50% of serum calcium, determining the rate of circulating active calcium. Another feature strictly related to calcium concentration is the volemic state of the patients. For this, we evaluated sodium, hemoglobin, and hematocrit as indirect indices of fluid volume. Hemoglobin and hematocrit are both related to serum calcium. For this reason, we decided to compute the ePVs, which considers both hemoglobin and hematocrit, as an indicator of volume state [11]. However, the relationship between NT-proBNP and serum calcium was significant despite the fact that the estimated PV status was added into the multivariate models as a confounding variable. Reduced renal function is often related to low calcium levels. It is mostly caused by binding to serum phosphate—commonly higher in CKD than in non-CKD patients—and lower vitamin D. Furthermore, the relationship between chronic renal damage and heart failure is well known in both directions. Indeed, CKD seems to impair heart function due to a worsening of the hypertension state, which increases vascular peripheral resistance and high chronic inflammation states, whereas heart failure and diastolic damage seem to accelerate renal damage [12,13].

BNP is a key mediator in cardiorenal syndrome. During cardiac dysfunction, such as heart failure, hypertension, or volume overload, elevated BNP levels promote diuresis and natriuresis, thereby reducing blood volume and alleviating cardiac stress. Conversely, renal hypoperfusion activates the renin–angiotensin–aldosterone (RAA) axis and facilitates sodium and water retention, increasing preload and stimulating BNP release to restore hemodynamic balance. Furthermore, BNP modulates peripheral vascular resistance and suppresses sympathetic nervous system activity, optimizing the intricate interplay between cardiac and renal function [6].

One of the major limitations of our study is the lack of urine calcium. As this study featured retrospective enrollment, urine calcium was present in only 4% of the data, and for this reason, it could not be evaluated. Ionized calcium is not available in our database, but the serum calcium-adjusted model included albumin as a covariate. Another limitation is the lack of parathormone dosage. There were no PTH doses closest to NT-proBNP. For this reason, this was not evaluated. Furthermore, our analysis included patients admitted in only one center in central Sicily, thus impairing the generalizability of our study. The main strengths are the large sample size and the longitudinal design. Furthermore, to our knowledge, no other studies have directly evaluated the association between nt-proBNP and serum calcium.

## 5. Conclusions

In conclusion, in our study, performed in a large cohort of patients, we found that NT-proBNP increases are linearly related to a reduction in calcium concentration. This result seems to be independent of personal hemoconcentration. albumin, phlogosis, and age. Further studies are needed to confirm this association, adding information on urine calcium to evaluate calcium excretion.

## Figures and Tables

**Table 1:** Baseline features of our sample.

Variables	Sample (n = 688)
NT-proBNP, pg/mL	3820 (1013–12,258)
Age, year	79 ± 12
Albumin, g/dL	3.1 ± 0.6
Calcium, mg/dL	8.5 ± 0.9
CRP, mg/dL	2.8 (0.8–7.1)
eGFR, mL/min/1.73 mq	54 (29–82)
Hb, g/dL	11.3 ± 2.3
Hct, %	34 ± 7
Potassium, mmol/L	4 ± 0.7
Sodium, mmol/L	139 ± 6
WBC, cc/mmc	8260 (6200–10,900)

CRP = C-reactive protein; eGFR = estimated glomerular filtration Rate; Hb = hemoglobin; Hct = hematocrit; WBC = white blood cell. Data are reported as mean standard deviation or median (interquartile range)

**Table 2:** Adjusted relationship between serum calcium and NT-proBNP.

Variables	Linear Regression		Linear Mixed Model	
	Adjβ	*p*-Value	Adjβ	*p*-Value
NTproBNP, pg/mL	−6.1 × 10^−7^	0.10	−6.9 × 10^−6^	0.01
Age, years	6.5 × 10^−5^	0.77	1.6 × 10^−3^	0.65
Albumin, g/dL	9.3 × 10^−2^	<0.01	7.3 × 10^−1^	<0.01
CRP, mg/dL	3.6 × 10^−4^	0.57	3.4 × 10^−3^	0.49
eGFR, mL/min/1.73 mq	−1.3 × 10^−4^	0.46	−1.5 × 10^−3^	0.29
ePVs, mL/g	−4.9 × 10^−3^	0.03	−4.0 × 10^−2^	0.03
Potassium, mmol/L	1.5 × 10^−2^	0.01	1.2 × 10^−1^	0.01

CRP = C-reactive protein; eGFR = estimated glomerular filtration rate; ePVs = estimated plasma volume state.

## Data Availability

The data presented in this study are available on request from the corresponding author. The data were not retrieved from a public repository but are stored in the informatic repository of our hospital.
